# *N*′-(5-Bromo-2-hy­droxy­benzyl­idene)-4-methyl­benzohydrazide

**DOI:** 10.1107/S1600536811043960

**Published:** 2011-10-29

**Authors:** De-Suo Yang

**Affiliations:** a Department of Chemistry and Chemical Engineering, Baoji University of Arts and Sciences, Baoji 721007, People’s Republic of China

## Abstract

The mol­ecule of the title compound, C_15_H_13_BrN_2_O_2_, displays an *E* conformation with respect to the C=N double bond and the dihedral angle between the planes of the benzene rings is 3.1 (2)°. An intra­molecular O—H⋯N inter­action generates an *S*(6) ring. In the crystal, mol­ecules are linked by N—H⋯O hydrogen bonds, forming *C*(4) chains along the *c*-axis direction.

## Related literature

For a related structure and background references, see: Yang (2008 ▶). For reference bond lengths, see: Allen *et al.* (1987 ▶).
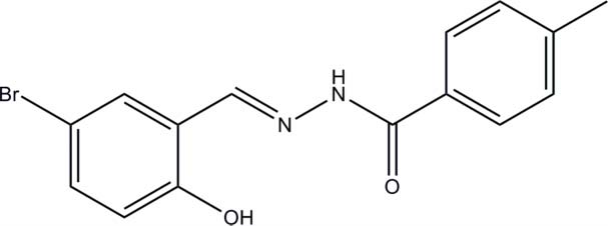


## Experimental

### 

#### Crystal data

C_15_H_13_BrN_2_O_2_
*M* *_r_* = 333.18
Monoclinic, 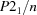

*a* = 5.8290 (15) Å
*b* = 31.914 (3) Å
*c* = 7.6440 (11) Å
β = 91.535 (2)°
*V* = 1421.5 (4) Å^3^
*Z* = 4
Mo *K*α radiation
μ = 2.89 mm^−1^
*T* = 298 K
0.27 × 0.23 × 0.23 mm

#### Data collection

Bruker SMART CCD diffractometer
Absorption correction: multi-scan (*SADABS*; Bruker, 2002 ▶) *T*_min_ = 0.509, *T*_max_ = 0.556
11208 measured reflections
3095 independent reflections
1794 reflections with *I* > 2σ(*I*)
*R*_int_ = 0.039

#### Refinement

*R*[*F* ^2^ > 2σ(*F* ^2^)] = 0.060
*wR*(*F* ^2^) = 0.147
*S* = 1.03
3095 reflections
186 parameters
1 restraint
H atoms treated by a mixture of independent and constrained refinement
Δρ_max_ = 0.90 e Å^−3^
Δρ_min_ = −0.77 e Å^−3^

### 

Data collection: *SMART* (Bruker, 2002 ▶); cell refinement: *SAINT* (Bruker, 2002 ▶); data reduction: *SAINT*; program(s) used to solve structure: *SHELXS97* (Sheldrick, 2008 ▶); program(s) used to refine structure: *SHELXL97* (Sheldrick, 2008 ▶); molecular graphics: *SHELXTL* (Sheldrick, 2008 ▶); software used to prepare material for publication: *SHELXTL*.

## Supplementary Material

Crystal structure: contains datablock(s) global, I. DOI: 10.1107/S1600536811043960/hb6461sup1.cif

Structure factors: contains datablock(s) I. DOI: 10.1107/S1600536811043960/hb6461Isup2.hkl

Supplementary material file. DOI: 10.1107/S1600536811043960/hb6461Isup3.cml

Additional supplementary materials:  crystallographic information; 3D view; checkCIF report

## Figures and Tables

**Table 1:** Hydrogen-bond geometry (Å, °)

*D*—H⋯*A*	*D*—H	H⋯*A*	*D*⋯*A*	*D*—H⋯*A*
O1—H1⋯N1	0.82	1.94	2.653 (5)	146
N2—H2⋯O2^i^	0.90 (1)	2.00 (2)	2.856 (5)	159 (5)

Symmetry code: (i) 
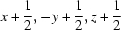
.

**Fig. 1.**
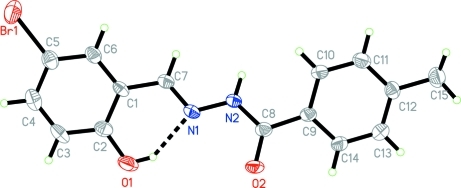
The structure of the title compound showing displacement ellipsoids drawn at the 30% probability level. Hydrogen bond is shown as a dashed line.

**Fig. 2.**
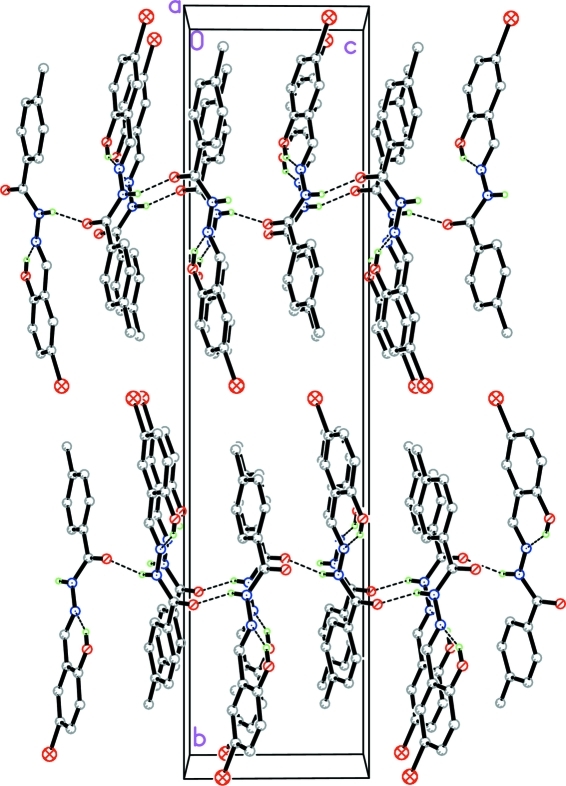
Molecular packing as viewed along the a axis. Hydrogen bonds are shown as dashed lines.

## supplementary crystallographic information

### Comment

As part of our ongoing studies of hydrazone compounds (Yang, 2008), the crystal structure of the title new hydrazone compound is reported.

In the title compound, Fig. 1, the molecule displays an *E* configuration with respect to the C═N double bond. The two benzene rings form a dihedral angle of 3.1 (2)°. All the bond lengths are within normal ranges (Allen *et al.*, 1987). The C7═N1 bond length of 1.267 (5) Å, conforms to the value for a double bond. The bond length of 1.346 (6) Å between atoms C8 and N2, is intermediate between a C—N single bond and a C═N double bond, because of conjugation effects in the molecule.

In the crystal structure, molecules are linked through N—H···O hydrogen bonds (Table 1), forming chains along the *c* axis (Fig. 2).

### Experimental

5-Bromo-2-hydroxybenzaldehyde (0.1 mmol, 20.1 mg) and 4-methylbenzohydrazide (0.1 mmol, 15.0 mg) were dissolved in CHCl_3_ (10 ml). The mixture was stirred at room temperature to give a clear colorless solution. Colourless blocks of the title compound were formed by gradual evaporation of the solvent over a period of a week at room temperature.

### Refinement

Atom H2 was located in a difference Fourier map and refined isotropically, with N—H distance restrained to 0.90 (1) Å. Other H atoms were placed in idealized positions and constrained to ride on their parent atoms, with O—H distance of 0.82 Å, C—H distances of 0.93–0.96 Å, and with *U*_iso_(H) = 1.2*U*_eq_(C) and 1.5*U*_eq_(O1 and C15).

### Crystal data

**Table:**

C_15_H_13_BrN_2_O_2_	*F*(000) = 672
*M**_r_* = 333.18	*D*_x_ = 1.557 Mg m^−^^3^
Monoclinic, *P*2_1_/*n*	Mo *K*α radiation, λ = 0.71073 Å
Hall symbol: -P 2yn	Cell parameters from 2291 reflections
*a* = 5.8290 (15) Å	θ = 2.5–24.1°
*b* = 31.914 (3) Å	µ = 2.89 mm^−^^1^
*c* = 7.6440 (11) Å	*T* = 298 K
β = 91.535 (2)°	Block, colorless
*V* = 1421.5 (4) Å^3^	0.27 × 0.23 × 0.23 mm
*Z* = 4	

### Data collection

**Table:**

Bruker SMART CCD diffractometer	3095 independent reflections
Radiation source: fine-focus sealed tube	1794 reflections with *I* > 2σ(*I*)
graphite	*R*_int_ = 0.039
ω scans	θ_max_ = 27.0°, θ_min_ = 2.6°
Absorption correction: multi-scan (*SADABS*; Bruker, 2002)	*h* = −7→7
*T*_min_ = 0.509, *T*_max_ = 0.556	*k* = −40→36
11208 measured reflections	*l* = −9→9

### Refinement

**Table:**

Refinement on *F*^2^	Primary atom site location: structure-invariant direct methods
Least-squares matrix: full	Secondary atom site location: difference Fourier map
*R*[*F*^2^ > 2σ(*F*^2^)] = 0.060	Hydrogen site location: inferred from neighbouring sites
*wR*(*F*^2^) = 0.147	H atoms treated by a mixture of independent and constrained refinement
*S* = 1.03	*w* = 1/[σ^2^(*F*_o_^2^) + (0.0437*P*)^2^ + 2.875*P*] where *P* = (*F*_o_^2^ + 2*F*_c_^2^)/3
3095 reflections	(Δ/σ)_max_ = 0.001
186 parameters	Δρ_max_ = 0.90 e Å^−^^3^
1 restraint	Δρ_min_ = −0.77 e Å^−^^3^

### Special details

**Table:**

Geometry. All e.s.d.'s (except the e.s.d. in the dihedral angle between two l.s. planes) are estimated using the full covariance matrix. The cell e.s.d.'s are taken into account individually in the estimation of e.s.d.'s in distances, angles and torsion angles; correlations between e.s.d.'s in cell parameters are only used when they are defined by crystal symmetry. An approximate (isotropic) treatment of cell e.s.d.'s is used for estimating e.s.d.'s involving l.s. planes.
Refinement. Refinement of *F*^2^ against ALL reflections. The weighted *R*-factor *wR* and goodness of fit *S* are based on *F*^2^, conventional *R*-factors *R* are based on *F*, with *F* set to zero for negative *F*^2^. The threshold expression of *F*^2^ > σ(*F*^2^) is used only for calculating *R*-factors(gt) *etc*. and is not relevant to the choice of reflections for refinement. *R*-factors based on *F*^2^ are statistically about twice as large as those based on *F*, and *R*- factors based on ALL data will be even larger.

### Fractional atomic coordinates and isotropic or equivalent isotropic displacement parameters (Å^2^)

**Table:**

	*x*	*y*	*z*	*U*_iso_*/*U*_eq_	
Br1	0.79558 (13)	0.008223 (19)	0.78795 (10)	0.0890 (3)	
N1	0.6602 (6)	0.20499 (12)	0.6325 (5)	0.0457 (9)	
N2	0.7868 (7)	0.24095 (12)	0.6598 (5)	0.0495 (10)	
O1	0.2871 (5)	0.16182 (12)	0.5406 (5)	0.0637 (9)	
H1	0.3660	0.1829	0.5528	0.096*	
O2	0.5871 (6)	0.27644 (10)	0.4522 (4)	0.0613 (9)	
C1	0.6256 (7)	0.13181 (14)	0.6772 (5)	0.0414 (10)	
C2	0.4073 (8)	0.12798 (16)	0.5996 (6)	0.0490 (11)	
C3	0.3068 (9)	0.08853 (18)	0.5819 (7)	0.0621 (14)	
H3	0.1613	0.0861	0.5300	0.075*	
C4	0.4188 (10)	0.05350 (18)	0.6397 (7)	0.0643 (14)	
H4	0.3497	0.0273	0.6280	0.077*	
C5	0.6353 (9)	0.05702 (16)	0.7156 (6)	0.0542 (12)	
C6	0.7349 (8)	0.09550 (15)	0.7348 (6)	0.0485 (11)	
H6	0.8800	0.0974	0.7878	0.058*	
C7	0.7427 (8)	0.17169 (14)	0.6988 (6)	0.0460 (11)	
H7	0.8808	0.1728	0.7625	0.055*	
C8	0.7419 (7)	0.27557 (14)	0.5644 (6)	0.0432 (10)	
C9	0.8885 (7)	0.31259 (13)	0.6038 (5)	0.0405 (10)	
C10	1.1045 (7)	0.30975 (15)	0.6837 (6)	0.0470 (11)	
H10	1.1659	0.2836	0.7120	0.056*	
C11	1.2287 (8)	0.34575 (16)	0.7212 (6)	0.0534 (12)	
H11	1.3736	0.3434	0.7742	0.064*	
C12	1.1425 (8)	0.38513 (15)	0.6819 (6)	0.0476 (11)	
C13	0.9283 (8)	0.38744 (15)	0.6002 (6)	0.0507 (12)	
H13	0.8673	0.4135	0.5707	0.061*	
C14	0.8044 (8)	0.35190 (14)	0.5619 (6)	0.0471 (11)	
H14	0.6609	0.3543	0.5066	0.056*	
C15	1.2769 (9)	0.42404 (17)	0.7256 (7)	0.0656 (15)	
H15A	1.2479	0.4323	0.8437	0.098*	
H15B	1.2311	0.4461	0.6469	0.098*	
H15C	1.4377	0.4185	0.7141	0.098*	
H2	0.897 (7)	0.2419 (17)	0.744 (5)	0.079*	

### Atomic displacement parameters (Å^2^)

**Table:**

	*U*^11^	*U*^22^	*U*^33^	*U*^12^	*U*^13^	*U*^23^
Br1	0.1148 (6)	0.0476 (3)	0.1037 (6)	0.0021 (3)	−0.0128 (4)	−0.0034 (3)
N1	0.041 (2)	0.051 (2)	0.044 (2)	−0.0050 (18)	−0.0146 (17)	−0.0014 (19)
N2	0.053 (2)	0.046 (2)	0.048 (2)	−0.0066 (18)	−0.0245 (18)	0.0062 (18)
O1	0.0426 (19)	0.080 (3)	0.067 (2)	−0.0050 (18)	−0.0158 (17)	0.002 (2)
O2	0.068 (2)	0.053 (2)	0.060 (2)	0.0059 (17)	−0.0395 (18)	−0.0024 (16)
C1	0.038 (2)	0.055 (3)	0.031 (2)	−0.010 (2)	−0.0030 (19)	−0.005 (2)
C2	0.048 (3)	0.063 (3)	0.036 (2)	−0.001 (2)	−0.001 (2)	0.000 (2)
C3	0.049 (3)	0.081 (4)	0.056 (3)	−0.026 (3)	−0.007 (2)	−0.009 (3)
C4	0.073 (4)	0.060 (3)	0.060 (3)	−0.024 (3)	0.002 (3)	−0.007 (3)
C5	0.061 (3)	0.057 (3)	0.045 (3)	−0.008 (2)	0.001 (2)	−0.008 (2)
C6	0.054 (3)	0.054 (3)	0.037 (3)	−0.009 (2)	−0.006 (2)	−0.005 (2)
C7	0.045 (3)	0.051 (3)	0.041 (3)	−0.003 (2)	−0.012 (2)	0.001 (2)
C8	0.045 (3)	0.046 (3)	0.038 (2)	0.006 (2)	−0.006 (2)	−0.004 (2)
C9	0.045 (2)	0.047 (3)	0.029 (2)	0.001 (2)	−0.0058 (19)	0.0038 (19)
C10	0.043 (3)	0.050 (3)	0.048 (3)	0.005 (2)	−0.007 (2)	0.007 (2)
C11	0.044 (3)	0.066 (3)	0.050 (3)	−0.004 (2)	−0.006 (2)	0.007 (3)
C12	0.053 (3)	0.052 (3)	0.038 (3)	−0.012 (2)	0.004 (2)	0.003 (2)
C13	0.057 (3)	0.046 (3)	0.049 (3)	0.006 (2)	0.002 (2)	0.004 (2)
C14	0.051 (3)	0.047 (3)	0.042 (3)	0.002 (2)	−0.008 (2)	0.005 (2)
C15	0.071 (4)	0.058 (3)	0.067 (4)	−0.015 (3)	−0.003 (3)	0.000 (3)

### Geometric parameters (Å, °)

**Table:**

Br1—C5	1.891 (5)	C6—H6	0.9300
N1—C7	1.267 (5)	C7—H7	0.9300
N1—N2	1.377 (5)	C8—C9	1.484 (6)
N2—C8	1.346 (6)	C9—C14	1.381 (6)
N2—H2	0.899 (10)	C9—C10	1.388 (6)
O1—C2	1.358 (6)	C10—C11	1.384 (6)
O1—H1	0.8200	C10—H10	0.9300
O2—C8	1.228 (5)	C11—C12	1.384 (7)
C1—C6	1.389 (6)	C11—H11	0.9300
C1—C2	1.395 (6)	C12—C13	1.383 (6)
C1—C7	1.452 (6)	C12—C15	1.501 (7)
C2—C3	1.394 (7)	C13—C14	1.372 (6)
C3—C4	1.362 (7)	C13—H13	0.9300
C3—H3	0.9300	C14—H14	0.9300
C4—C5	1.379 (7)	C15—H15A	0.9600
C4—H4	0.9300	C15—H15B	0.9600
C5—C6	1.365 (6)	C15—H15C	0.9600
			
C7—N1—N2	116.3 (3)	O2—C8—C9	122.1 (4)
C8—N2—N1	120.4 (3)	N2—C8—C9	116.2 (4)
C8—N2—H2	119 (4)	C14—C9—C10	118.2 (4)
N1—N2—H2	120 (3)	C14—C9—C8	118.5 (4)
C2—O1—H1	109.5	C10—C9—C8	123.2 (4)
C6—C1—C2	117.9 (4)	C11—C10—C9	120.0 (4)
C6—C1—C7	119.0 (4)	C11—C10—H10	120.0
C2—C1—C7	123.1 (4)	C9—C10—H10	120.0
O1—C2—C3	118.3 (4)	C12—C11—C10	121.6 (4)
O1—C2—C1	121.9 (4)	C12—C11—H11	119.2
C3—C2—C1	119.8 (5)	C10—C11—H11	119.2
C4—C3—C2	120.9 (5)	C13—C12—C11	117.7 (4)
C4—C3—H3	119.6	C13—C12—C15	121.1 (5)
C2—C3—H3	119.6	C11—C12—C15	121.2 (4)
C3—C4—C5	119.6 (5)	C14—C13—C12	121.0 (4)
C3—C4—H4	120.2	C14—C13—H13	119.5
C5—C4—H4	120.2	C12—C13—H13	119.5
C6—C5—C4	120.0 (5)	C13—C14—C9	121.3 (4)
C6—C5—Br1	120.3 (4)	C13—C14—H14	119.3
C4—C5—Br1	119.7 (4)	C9—C14—H14	119.3
C5—C6—C1	121.8 (4)	C12—C15—H15A	109.5
C5—C6—H6	119.1	C12—C15—H15B	109.5
C1—C6—H6	119.1	H15A—C15—H15B	109.5
N1—C7—C1	121.2 (4)	C12—C15—H15C	109.5
N1—C7—H7	119.4	H15A—C15—H15C	109.5
C1—C7—H7	119.4	H15B—C15—H15C	109.5
O2—C8—N2	121.7 (4)		

### Hydrogen-bond geometry (Å, °)

**Table:**

*D*—H···*A*	*D*—H	H···*A*	*D*···*A*	*D*—H···*A*
O1—H1···N1	0.82	1.94	2.653 (5)	146
N2—H2···O2^i^	0.90 (1)	2.00 (2)	2.856 (5)	159 (5)

Symmetry codes: (i) *x*+1/2, −*y*+1/2, *z*+1/2.
